# Grow Beautiful

**Published:** 1857-03

**Authors:** 


### GROW BEAUTIFUL.

PERSONS may outgrow disease, and become healthy, by proper attention to the laws of their physical constitutions. By moderate and daily exercise, men may become active and strong in limb and muscle. But to grow beautiful, how? Age dims the lustre of the eye, and pales the roses on beauty's cheek; while crow-feet, and furrows, and wrinkles, and lost teeth, and gray hairs, and bald head, and tottering limbs, and limping feet, most sadly mar the human form divine. But dim as the eye is, as pallid and sunken as may be the face of beauty, and frail and feeble that once strong, erect, and manly body, the immortal soul, just fledging its wings for its home in heaven, may look out through these faded windows as beautiful as the dewdrops of a summer's morning, as melting as the tear that glistens in affection's eye—by growing kindly, by cultivating sympathy with all human kind; by cherishing forbearance towards the foibles and follies of our race, and feeding day by day on that *love to God and man* which lifts us from the brute, and makes us akin to angels.

